# The Child Healthcare at MATER Pediatric Study (CHAMPS): a 2-arm cluster randomized control trial of group well child care for mothers in treatment for opioid use disorder and their children

**DOI:** 10.1186/s13063-023-07357-2

**Published:** 2023-05-17

**Authors:** Vanessa L. Short, Diane J. Abatemarco, Erica Sood, Dennis J. Hand, Meghan Gannon, Jobayer Hossain, Neera K. Goyal

**Affiliations:** 1 Thomas Jefferson University, 1233 Locust Street Suite 401, Philadelphia, PA USA; 2 Nemours Children’s Health, 1600 Rockland Rd, Wilmington, DE USA; 3 Department of Pediatrics, Thomas Jefferson University, 833 Chestnut Street, Philadelphia, PA USA

**Keywords:** Group well child care, Opioid use disorder

## Abstract

**Background:**

Studies suggest that group-based well child care—a shared medical appointment where families come together as a group to receive pediatric primary care—increases patient-reported satisfaction and adherence to recommended care. Evidence supporting the use of group well child care for mothers with opioid use disorder, however, is lacking. The overall objective of the Child Healthcare at MATER Pediatric Study (CHAMPS) trial is to evaluate a group model of well child care for mothers with opioid use disorder and their children.

**Methods:**

CHAMPS is a single-site 2-arm cluster randomized controlled trial. A total of 108 mother–child dyads will be enrolled into the study. Twenty-six clusters of approximately 4 mother-infant dyads each will be randomized 1:1 to one of two study arms (intervention or control). Clustering will be based on child’s month of birth. In the intervention arm, group well child care will be provided on-site at a maternal substance use disorder treatment program. Mother–child dyads in the control arm will receive individual well child care from one nearby pediatric primary care clinic. Dyads in both study arms will be followed prospectively for 18 months, and data will be compared between the two study arms. Primary outcomes include well child care quality and utilization, child health knowledge, and parenting quality.

**Discussion:**

The CHAMPS trial will provide evidence to determine if a group well child care offered on-site at an opioid treatment program for pregnant and parenting women is beneficial over individual well child care for families impacted by maternal opioid use disorder.

**Trial registration:**

ClinicalTrials.gov identifier: NCT05488379. Registered on Aug. 04, 2022.

## Administrative information

Note: the numbers in curly brackets in this protocol refer to SPIRIT checklist item numbers. The order of the items has been modified to group similar items see http://www.equator-network.org/reporting-guidelines/spirit-2013-statement-defining-standard-protocol-items-for-clinical-trials/).

**Table:** 

Title {1}	The Child Healthcare at MATER Pediatric Study (CHAMPS): a 2-arm cluster randomized control trial comparing group well child care to individual well child care for mothers in treatment for opioid use disorder and their children
Trial registration {2a and 2b}	ClinicalTrials.gov identifier: NCT05488379 All items from the World Health Organization trial registration data set can be found within the study’s protocol
Protocol version {2}	January 5, 2023
Funding {3}	Agency for Healthcare Research and Quality (project number 1R18HS027399-01)
Author details {5a}	^a^ Thomas Jefferson University 1233 Locust Street Suite 401 Philadelphia, PA, USA Vanessa.Short@jefferson.edu Meghan.Gannon@jefferson.edu Dennis.Hand@jefferson.edu Diane.Abatemarco@jefferson.edu ^b^ Nemours Children’s Health 1600 Rockland Rd Wilmington, DE Erica.Sood@nemours.org Neera.Goyal@nemours.org Jobayer.Hussein@nemours.org ^c^ Department of Pediatrics Thomas Jefferson University 833 Chestnut Street Philadelphia, PA, USA Neera.Goyal@nemours.org
Name and contact information for the trial sponsor {5b}	Agency for Healthcare Research and Quality
Role of sponsor {5c}	The funding agency was not involved in the study design; in the collection, analysis, and interpretation of data; in the writing of the report; and in the decision to submit the article for publication.

## Introduction

### Background and rationale {6a}

In the past two decades, the rate of opioid use disorder (OUD) among pregnant women has more than quadrupled, constituting a US public health crisis [1–3]. Perinatal OUD presents a unique set of healthcare challenges for affected mothers and their children across a continuum of care. For this population, multiple clinical and psychosocial risk factors contribute to increased rates of pregnancy complications, newborn morbidity, and childhood developmental and health concerns. Although most research on perinatal OUD has focused on pregnancy and birth hospitalization care, fewer studies have focused on pediatric primary care after discharge from the hospital, when ongoing risks associated with OUD may include a myriad of factors including fear of stigma, low health literacy, maladaptive parenting behaviors, and economic and social barriers to seeking healthcare.

Addressing social and emotional determinants of health is a core focus of pediatric primary care clinicians, particularly in the context of well child care (WCC) visits. WCC visits, which occur at age-specific intervals defined by the American Academy of Pediatrics, emphasize anticipatory guidance, psychosocial screening, connection to community resources, and assessments of child growth, health, development, and behavior. There is evidence that WCC could be improved for children impacted by maternal OUD, who often experience multiple clinical and psychosocial concerns in infancy and early childhood such feeding difficulties, hepatitis C exposure, and developmental delays [4]. Despite these concerns, low attendance to WCC for this patient population has been reported [5], and mothers with OUD have described traditional WCC visits in the pediatric primary care setting as limited in time and resources to comprehensively address their questions and needs [6, 7]. Lack of time and resources to comprehensively address care needs of mothers with OUD and their children has also been reported by primary care pediatricians [8]. There is an urgent need for research to enhance the effectiveness, perceived value, and quality of pediatric primary care for this growing and underserved population.

One promising intervention for mothers in treatment for OUD and their children is group WCC, in which multiple mother–child dyads receive pediatric care together. Group WCC visits encourage increased breadth and depth of discussion, peer-to-peer support, and greater emphasis on self-care and behavior change. In addition to standard WCC topics focused on child health and development, the group WCC visit format places emphasis on maternal wellness including nutrition and exercise, family planning, stress reduction, and interpersonal relationships. Available research suggests this intervention increases patient-reported satisfaction and adherence to recommended care [9–13]. However, evidence supporting the use of group WCC for mothers with OUD is lacking.

### Objectives {7}

The overall objective of this trial is to evaluate a group model of WCC for mothers with OUD and their children.

### Trial design {8}

This is a single-site cluster randomized controlled trial. One hundred eight pregnant and parenting women receiving treatment for OUD from one single opioid treatment program will be enrolled into the study. Twenty-six consecutive clusters—based on the one-month birth intervals of participants’ infants—of ~ 4 dyads will be randomized in a 1:1 ratio to one of two study arms. Thirteen clusters will be randomized to individual WCC (control group), and 13 birth clusters will be randomized to group WCC (intervention arm), with concealment of randomization from participants until after they have completed their initial enrollment study visit, or, for those who are pregnant at time of enrollment, immediately following delivery. Randomization by birth month was selected to ensure a close age range among infants receiving group WCC together in the intervention arm. Blocking or stratification was not used in the randomization. Dyads in both study arms will be followed prospectively for 18 months, and data will be compared between the two study arms. Primary outcomes include well child care quality and utilization, child health knowledge, and parenting quality. We hypothesize that at the end of the study period, participants in the intervention arm will have increased WCC engagement, knowledge about parenting, and maternal responsiveness compared to participants in the control arm.

## Methods: participants, interventions, and outcomes

### Study setting {9}

This trial is conducted within a single, urban substance use disorder (SUD) treatment center affiliated with a university and university health system. The treatment center includes a licensed outpatient opioid treatment program and a residential treatment program, both of which serve pregnant and/or parenting women exclusively. At both outpatient and residential levels of care, all patients receive comprehensive wraparound services, including methadone and buprenorphine as medications for OUD, psychiatric medications and psychotherapy, cognitive behavioral therapy and mindfulness-based therapy for substance use disorder in individual, family, and group formats, case management, medical care coordination, peer recovery support, mindfulness-based parenting psychoeducation, and various other services especially needed by the population of pregnant and/or parenting women with SUDs. At the residential level of care, women live with their children at the facility and receive the majority of programming at the facility for an average of 6 months. Upon completion of residential treatment, the majority of patients continue their SUD treatment at the outpatient facility. Patients are welcome to participate in the outpatient program for as long as they desire, and the average length of outpatient treatment is 3–4 years. Across both programs, the treatment center serves approximately 250 women each year.

### Eligibility criteria {3}

Inclusion criteria for screening for and consent into the trial are as follows: (1) 18 years or older, (2) at least 28 weeks pregnant or parenting a child less than 12 months of age, (3) receiving treatment for OUD from the recruitment opioid treatment program, (4) ability to read and speak English, (5) plans to parent infant after delivery (if pregnant), (6) intent to seek pediatric care within the affiliated health system, and (7) intent to remain in the local area for the next 2 years. Exclusion criteria include (1) inability to speak English, (2) plans to move outside of the local area in the next 2 years, (3) plans to not parent infant after delivery, and (4) plans to seek pediatric care outside of the affiliated health system.

### Who will take informed consent? {26a}

A 2-page study flyer is posted at the check-in desks of the treatment program’s outpatient and residential facilities. This flyer explains the study and provides contact information for the research coordinator and principal investigators. Additionally, research personnel review the opioid treatment program’s pregnancy census list weekly for new pregnant admissions or newly delivered clients. Staff at the opioid treatment program are asked to identify eligible individuals. All identified individuals are contacted by study personnel, either by phone or in-person, informed about the study, and asked if they are willing to participate. If they agree, an enrollment interview is carried out. Study personnel explain the purpose of the study, answer questions, and obtain written informed consent from those who meet study inclusion criteria. Those who consent to participate are provided with a copy of the consent form with contact details of the research team. Eligible individuals who do not agree to participate are asked to self-report basic sociodemographic information (e.g., age, education level, number of kids, and reason for refusal) to allow a comparison of enrolled and non-enrolled individuals. All staff responsible for obtaining consent are trained and certified in the protection of human subjects and study-specific consent procedures.

### Additional consent provisions for collection and use of participant data and biological specimens {26b}

When obtaining informed consent, participants are informed that their data will be de-identified and stored in a secure database and that any data released will not be identifiable. Participants will be told they can withdraw consent at any time during the trial. This trial does not involve collecting biological specimens. Data will not be used or distributed for future research studies.

## Interventions

### Explanation for the choice of comparators {6b}

The intervention is an adapted version of an existing, evidence-based model of group WCC (*CenteringParenting *[14]). Standard individual WCC is the comparator.

### Intervention description {11a}

As the framework for the group WCC intervention, we leveraged Centering®, a model of care delivery developed by The Centering® Healthcare Institute [15]. Centering® seeks to shift the paradigm of health services from individual to group care, bringing patients out of a traditional office and into a group setting where they learn from their providers and each other. Each group session has planned core content, with consistency of providers across all sessions, and 2 h per session for health assessment, interactive learning, and community building. Instead of didactic education, providers use facilitative leadership, encouraging patients to fully engage in their care and raise issues of importance to them.

*CenteringParenting*is the pediatric adaptation of Centering® and is based on the American Academy of Pediatrics recommended schedule of visit periodicity, including developmental milestone assessments, anticipatory guidance, and immunizations [16]. In addition to standard WCC topics focused on infant health and development, *CenteringParenting*places emphasis on maternal wellness including nutrition and exercise, family planning, stress reduction, and interpersonal relationships [14, 17].

Participants who enroll into CHAMPS during pregnancy and are randomized to the intervention arm initially receive routine individualized well care after birth hospital discharge. This consists of an initial 30-min newborn visit usually within 2–3 days after discharge at the affiliated pediatric primary care office, with any additional 15-min visits in the first few weeks of life as needed, i.e., for jaundice or a weight check. Beginning with the 1-month WCC visit, mother-infant dyads grouped by birth month (anticipated *n* = 3–5 per group) are scheduled for WCC together, with subsequent visits occurring at the American Academy of Pediatrics recommended intervals (2, 4, 6, 9, 12, 15, and 18 months of age). Group WCC visits occur on-site at the opioid treatment program. Infants with an extended hospitalization beyond 1 month are eligible to join into a later group session if needed.

Participants who are already parenting a child < 12 months of age at the time of enrollment and are randomized to the intervention arm are grouped with other mother-infant dyads by infant birth month and scheduled for WCC together. Group WCC visits occur on-site at the opioid treatment program. Visits occur at the American Academy of Pediatrics recommended intervals.

Group WCC sessions diverge from individual WCC in the following ways:

- *Setting and duration*: Group WCC sessions occur in a dedicated group room at the SUD treatment program and last 2 h. A room divider with a computer and an examination table offers a private area for pediatricians to examine the infant and review history, current issues, and other maternal concerns. The group space itself entails a circle of chairs or pillows on the floor with mats in the middle for infants. There are shelves with children’s books and toys that participants may use. Healthy snacks are provided during the session for the mothers and their children (when age appropriate).
- *Provider interaction*: Each group is assigned to one pediatrician who will lead all WCC sessions for that group, with a nurse practitioner from the opioid treatment program serving as co-facilitator. All providers involved in delivering group WCC are trained in group facilitation by Centering Healthcare Institute, which emphasizes skills such as reflective listening and strategic questioning as opposed to didactic education. A pediatric nurse assists with immunizations and other procedures.
- *Content*: A structured, reproducible modular curriculum guides session content, with flexibility based on group needs. Facilitated group discussions provide opportunity for skills building, knowledge sharing, and normalization of early parenting experiences. In addition to standard topics within the *CenteringParenting *curriculum, emphasis on specific clinical topics is tailored to the patient population. Topics were selected and prioritized based on the team’s collective experience as experts in OUD treatment and pediatric primary care and from results from the team’s formative qualitative research conducted with mothers with OUD and pediatric providers [7]. Aspects of Mindfulness Based Parenting [18, 19]—a relational approach developed from the principles of mindfulness and that calls for the full attention of parents when interacting with their children—is also incorporated into each session.
- *Scheduling*: Sessions are scheduled in advance to reoccur on consistent days and time in coordination with other scheduled groups at the opioid treatment program. Participants who no-show for group WCC are contacted to schedule a routine office-based individual visit. Participants in group WCC are able to schedule acute visits in the office as needed.

### Control description

Clusters randomized to the control arm receive routine individualized WCC at an urban, hospital-owned academic pediatric practice located 5 city blocks from the opioid treatment program. This practice site includes 14 physicians and nurse practitioners, serves a predominantly Medicaid-insured population, and is a training environment for students and residents. Newborns are scheduled for an initial 30-min visit within 3–5 days after discharge home, with any additional 15-min visits in the first few weeks of life as needed (i.e., for jaundice or a weight check). Subsequent 15-min WCC visits occur at recommended intervals per American Academy of Pediatrics recommendations (1, 2, 4, 6, 9, 12, 15, 18, 24, 30, and 36 months of age). Routine care in this practice setting includes maternal depression screening, provision of books as part of a national literacy promotion program, developmental screening, and social determinants of health screening. A dedicated RN coordinator tracks receipt of preventative services for all patients and provides outreach to patients who no-show for WCC, and access to subspecialty care, social work, and lactation services are available on-site. Scheduling of visits may occur in-person at the registration desk, through an online patient portal, or by telephone. Phone or text appointment reminders are provided the day before scheduled visits.

### Criteria for discontinuing or modifying allocated interventions {11b}

Patients can leave the study at any time for any reason if they wish to do so without any consequences. Following a live birth, participants will remain eligible to continue in the study unless the mother and/or infant experiences the following: gestational age at time of delivery less than or equal to 32 weeks or illness or clinical complication warranting prolonged hospitalization after delivery. Even when a participant randomized to the intervention does not receive the intervention, we will attempt to complete the follow-up data collection to ensure a complete dataset.

### Strategies to improve adherence to interventions {11c}

We will use multiple strategies to retain the study sample over the duration of the study. These strategies include (1) providing reminders calls before visits; (2) limiting participant burden by scheduling visits when participants are already attending the treatment program for medication dispensing or therapy; (3) paying for car ride services (e.g., Uber) and providing vouchers for parking lots for participants to use while they are completing study visits; and (4) sharing information about the study with opioid treatment program staff and providers across the hospital system (e.g., Neonatology, General Pediatrics, Ob/Gyn).

### Relevant concomitant care permitted or prohibited during the trial {11d}

No concomitant care or interventions are prohibited during the trial.

### Provisions for post-trial care {30}

There are no plans for any sort of provision for post-trial care. The last scheduled intervention visit occurs at approximately 18 months post-randomization. It is expected that healthcare providers who deliver the intervention will specify the need for additional WCC post-intervention for each child randomized to the intervention arm of the trial.

### Outcomes {12}

Primary outcomes were selected based on the hypothesized mechanism of effect for the intervention, importance to our target population, and published literature on group WCC in other populations.

#### Primary outcomes

1. *WCC engagement*: measured as both utilization and experience of WCC.
  - *WCC utilization*: The total number of age-appropriate WCC visits during the 18-month study period will be abstracted from the pediatric electronic medical record (EMR). Adequacy of WCC during this time will be dichotomized as “adequate” (completes the recommended number of WCC visits) or “inadequate” (does not complete the recommended number of WCC visits). This definition is adapted from the Health Plan Employer Data and Information Set (HEDIS) measure for WCC [20]. The mean number of age-appropriate WCC visits during the 18-month study period and the proportion of participants with adequate WCC over the 18-month study period will be compared between the 2 study arms.
  - *WCC experience*: The *Promoting Healthy Development Survey* (PHDS) [21], a 43-item standardized, validated parent survey designed to measure the quality and content of care for children 3–48 months, is completed by participants at the 6-, 12-, and 18-month follow-up assessment visits. The PHDS will be used to evaluate participants’ perceptions of their child’s WCC, including (1) anticipatory guidance—whether the mother received age-appropriate guidance; (2) developmental surveillance—whether the mother was asked about concerns about her child’s learning, development, or behavior, and if concerns were addressed; (3) family-centeredness—whether the mother felt her perspective was heard and valued; (4) family risk assessment—whether the mother was asked about issues in the family (e.g., substance use, emotional support); and (5) helpfulness of care—how helpful the care provided was in specific aspects of parenting (e.g., understanding child behavior). Multiple survey items are used to calculate a score for each component of care evaluated and these composite measures have been previously tested for internal consistency. The mean score for each component of care at each time point will be compared between the 2 study arms.
2. *Parenting knowledge*: The medical literature was reviewed for previously validated questions used to assess parental knowledge about the care and development of intrauterine opioid-exposed infants; no prior questions were identified. Therefore, a survey instrument includes investigator-developed questions specific to this area, (e.g., “Breastfeeding increases the chance your baby will have opioid withdrawal symptoms: Agree/Disagree/Not Sure”), as well as questions from the Knowledge of Infant Development Inventory (KIDI) [22]. The KIDI is a widely used measure of parenting knowledge designed as a diagnostic tool for high-risk parents. The KIDI consists of 58 items grouped into four categories (norms and milestones, principles, parenting, and health and safety) that tests mothers’ knowledge of children’s development and parenting of children from birth through 36 months of age. Items are measured on a 5-point Likert scale with higher scores indicating correct knowledge and developmentally appropriate expectations. In addition to a total knowledge score calculated by summing the number of correct items and calculating an average, four KIDI subscale scores and a knowledge of infant opioid exposure score will also be generated. The total and subscale score at 18 months will be compared between the 2 study arms. The mean change in scores from baseline to 18 months follow-up will be compared between the two study arms.
3. *Maternal-child interaction quality*: This is directly assessed using video-recorded mother/child free play at an 18-month follow-up assessment visit. Interactions are coded by certified scorers using the Keys to Interactive Parenting Scale (KIPS) [23]. KIPS assesses 12 parenting behaviors using quality ratings ranging from 1 to 5 within three domains sub-scale domains: building relationships, promoting learning engagement in language experiences, and supporting confidence. A total score is generated by summing the 12 items and calculating an average to indicate low, moderate, and high-quality parenting behavior. The total and subscale score at 18 months will be compared between the 2 study arms.

#### Secondary outcomes

##### Infant healthcare utilization

Data are abstracted from the child’s EMR at the end of the 18-month follow-up period, including receipt of on-time immunizations and screenings (i.e., lead, hemoglobin, and developmental assessments); infant hepatitis C screening at 18 months among those with perinatal hepatitis C exposure; the number of acute primary care visits (“sick visits”); emergency department utilization and hospitalizations; subspecialist referrals and visits (e.g., Neurology, Ophthalmology, Developmental Medicine); and early intervention referral and enrollment. The proportion of participants who receive these services within the 18-month study period will be compared between the 2 study arms. The mean number of hospitalizations and sick, emergency, and specialist visits will also be compared between the 2 groups.

##### Infant development

This is self-reported at the 6-, 12-, and 18-month follow-up assessment visit using Ages & Stages Questionnaires, 3rd Edition (ASQ-3), a set of 40-item parent-reported age-specific developmental screening questions assessing communication, gross motor, fine motor, problem-solving, and personal-social skills [24]. Domain-specific scores are calculated. Mean ASQ-3 scores, abnormal ASQ-3 scores, and the number of children with any abnormal ASQ-3 scores will be compared between the 2 study arms.

##### Maternal health behaviors

Participants who enroll into CHAMPS while pregnant self-report: duration of breastfeeding at 1, 6, and 12 months; infant sleep positioning at 1, 6, and 12 months; contraception use at 1, 6, and 12 months; and repeat pregnancy within 12 months of follow-up. The proportion of participants who report these behaviors will be compared between the 2 study arms.

##### Maternal psychosocial

This is self-reported at the 1-, 6-, 12-, and 18-month follow-up assessment visits and includes depression using the Edinburg Postnatal Depression Scale [25]; general stress using the Perceived Stress Scale [26]; parenting stress using the Parenting Stress Index [27]; social support using the Social Provisions Scale [28]; and resilience using the Connor-Davidson Resilience Scale [29]. The total score and the subscale scores at 18 months, and the change in scores overtime will be compared between the 2 study arms.

##### Maternal illicit substance use

This will be defined as any and number of positive urine drug screens for illicit substances at the 6-, 12-, and 18-month follow-up assessment visits. Data will be abstracted from the opioid treatment program records. The proportion of participants with any positive screen and the mean number of positive screens at each time point will be compared between the 2 study arms.

##### Maternal sensitivity/responsiveness

This is self-reported at the 12-month follow-up assessment visit using the Maternal Responsiveness Questionnaire [30], which assesses the extent to which mothers engage with and respond to their infants. The measure yields three scored subscales (responsiveness, non-responsiveness, and delayed responsiveness). At the 18-month follow-up assessment visit, the Coping with Toddlers’ Negative Emotions Scale [31] is completed which measures the degree to which mothers perceive themselves as reactive to their children's negative affect in distressful situations. Six subscales are derived that reflect the specific types of coping response parents tend to use in such situations. Mean subscale scores for each questionnaire will be compared between the 2 study arms.

### Participant timeline {13}



### Sample size {14}

Assuming 150 births in the opioid treatment program over an 18-month recruitment period, > 90% rate of maternal legal guardianship at birth, 80% study participation rate, and equivalent group allocation (50%), we expect to enroll 108 participants (n = 54 per study arm) during the project period. A sample size of 54 per study arm will produce a two-sided 95% CI with a distance from the standardized difference in means between two arms to the limits that are equal to 0.38. Additionally, with a sample size of 54 per study arm, a two-sided test for the time-averaged difference between two means in a repeated measures design with a significance level of 0.05 will have 80% power to detect a standardized difference in means of 0.43 in a design with 4 repeated-measurements taken over the 18-month period and with between level correlations of 0.5. A sample size of 54 per study arm will give us 80% power to detect a standardized difference in means of 0.54 (effect size) between two arms using a two-sided two-sample t-test with a significance level of 0.05. Of note, we were unable to find reported data about the cluster effect of different birth months. However, we expect only a negligible cluster effect for birth-month, and so assumed no to negligible intra-cluster correlation coefficient (ICC; 0 to 0.05) to perform the power calculations, which is almost equivalent to performing simple random sampling. However, we preferred the design of cluster random sampling to simple random sampling to account for the effect of birth-month if there is any.

### Recruitment {15}

IRB-approved study flyers are posted in the opioid treatment program waiting and clinical rooms and instruct individuals interested in participating to notify study personnel to learn more about the study. Research personnel and treatment program staff also distribute study information to potentially eligible individuals. These individuals are approached subsequently by the study personnel and told about the study using a scripted verbal description of the study. All potential participants, regardless of how they learn about the study, are provided with detailed information about study procedures. Those who are subsequently screened, eligible to participate, and agree to participate are asked to provide written informed consent.

## Assignment of interventions: allocation

### Sequence generation {16a}

A complete random assignment procedure is used to allocate clusters to study arms. The package “randomizr” of the statistical software R was used for this assignment.

### Concealment mechanism {16b}

As an open-label study, both participants and researchers know about the assignment of participants to the treatment groups. The treatment assignment sequence was forwarded to the principal investigator of the project after generation.

### Implementation {16c}

The project statistician generated the treatment assignment sequence. The research coordinator informs participants to their allocation.

## Assignment of interventions: blinding

### Who will be blinded {17a}

Due to the nature of the study, participants and the investigator responsible for delivering group WCC are not blinded to group allocation. However, all participants are assigned a unique identification number at enrollment to ensure that research personnel responsible for analysis will be blinded to allocation status.

## Data collection and management

### Plans for assessment and collection of outcomes {18a}

Data is derived through EMR abstraction and from questionnaires completed during study assessment visits. Data from all sources are matched by the participant’s unique study identification number and entered into one master electronic REDCap database. Data entry and checking is an ongoing process as the data become available. The final analytic dataset will be void of personal identifiers to protect the confidentiality of participants.

1. *Medical record abstraction*: EMR data of participants and their children are abstracted by research personnel following participant enrollment into the study, the birth of a participant’s child, and at the 6-, 12-, and 18-month study visits. A standard data abstraction form is used.
2. *Participant assessments*: All randomized study participants in both study arms will have a total of 5 assessment visits with research personnel. The first visits will occur immediately following enrollment into the study (baseline). For participants who enroll while pregnant, subsequent study assessment visits will occur prior to the participant’s child turning 1 month of age, and within 4 weeks of the participant’s child turning 6, 12, and 18 months of age. For participants who are not pregnant when they enroll, subsequent study assessment visits will occur at 1, 6, 12, and 18 months post-enrollment. At each assessment visit, participants will complete a set of standardized questionnaires in a private research room using traditional paper-and-pencil forms. No personally identifying information will be collected on the questionnaires. The 18-month study visit will also involve a 15-min observed free play session between the mother and child, which will be videotaped and subsequently coded according to standardized procedures using the *Keys to Interactive Parenting Scale* [23].
3. *In-depth interviews*: Participants in the intervention arm will participate in semi-structured, individual in-person interviews at 9 and 18 months after their first group WCC session. These interviews will be conducted in a private research room, last 20–30 min, and be audio recorded and transcribed verbatim by a HIPAA compliant transcription service. During the session, participants will be asked to discuss things that they like and dislike about the group WCC program and how the program could be improved. Participants will be asked to report their experiences of receiving care in a group format, focusing on session structure, interaction with other mothers, and receiving specific information. Standardized interview question guides with prompts to engage participants in a fluid discussion will be used.

### Plans to promote participant retention and complete follow-up {18b}

Multiple strategies are used to retain participants over the duration of the study, including (1) providing reminders (e.g., calls, texts) before study data collection visits; (2) limiting participant burden by scheduling data collection study visits when participants are already attending the SUD treatment center; (3) using limited release of medical information forms for EMR abstraction; (4) providing incentives after completion of data collection visits; (5) paying for car ride services (e.g., Uber) and providing parking vouchers to nearby parking garages so participants can park during study visits; (6) collecting contact information of friends and family members who are likely to know the location of subjects should they be temporarily out of contact; and (7) sharing information about the study with staff and providers across hospital departments (e.g., Neonatology, General Pediatrics, Ob/Gyn). In addition, our approach to analyzing the data will allow us to include subjects in analyses who provide partial data due to premature study dropout. Comparisons between study subjects who are retained and who drop out will be conducted to determine biases and non-random patterns of study attrition.

### Data management {19}

Data will be collected primarily using paper-based forms. Research staff will be responsible for entering data from all completed paper form into a secure Research Electronic Data Capture (REDCap) database. Data quality validation with inbuilt quality checks will be programmed into REDCap.

### Confidentiality {27}

All participants will be assigned an anonymous study identification number at enrollment. Study IDs will be used on all instrumentation and will be linked to an individual’s identifying information by a master study key which will be maintained in hard copy locked in the principal investigator’s office. All hard copies of study files as well as videotapes of the recorded play sessions and audiotapes of the interview sessions and focus group discussions will be kept in locked cabinets in research personnel office. Data collection instruments (e.g., questionnaires, EMR abstraction tools), coded video data, and transcripts will omit names and identifiers that could potentially identify the participants. All electronic data files will be saved on a University password-protected, encrypted computer and be backed up daily. Documentation of data management procedures will be carefully maintained. No results will ever be reported in a personally identifiable manner.

Each participant randomized to group WCC will have the opportunity to privately discuss any concerns about her child with the pediatrician before the group visit begins. The group WCC model of care is designed to promote greater breadth and depth of discussion, as well as increased focus on maternal wellness and social determinants of health. Therefore, it is possible that group discussion may bring forth sensitive topics, for example, intimate partner violence that could cause psychological distress for participants. Prior to each group WCC visit, the pediatrician will review group rules including confidentiality and the use of the session as a “safe space.” Any participant experiencing significant distress as a result of the discussion will be escorted from the room by one of the co-facilitators, and an on-site nurse manager or social worker at the SUD treatment program will be notified. In the case information is disclosed during group discussions about child abuse or neglect, intent to harm self, or intent to harm others, the pediatrician will follow standard reporting procedures as required by law.

### Plans for collection, laboratory evaluation, and storage of biological specimens for genetic or molecular analysis in this trial/future use {33}

N/A

## Statistical methods

### Statistical methods for primary and secondary outcomes {20a}

#### Quantitative data

Analyses will follow intent-to-treat principles, with participants analyzed according to randomized treatment assignment of the cluster in which they reside. Most analyses will use the individual participant as the unit of analysis, but because randomization is at the cluster level, model-based approaches that account for the correlation between individual participants within a cluster will be used.

Collected data will be summarized in tabular and graphic forms. Demographics and other study variables will be summarized by treatment groups. Mean (SD) or median (interquartile), as appropriate, will be used to describe numerical data, and frequency and percentage will be used to describe the distribution of categorical variables between treatment groups. Two-sample t-test, Mann–Whitney U test, chi-square test, and Fisher’s exact test will be used to compare the distribution of study variables between two-intervention groups as appropriate. Longitudinally collected data will be summarized by intervention groups and data collection time points.

Analysis of variance (ANOVA) and generalized linear and logistic regression models will be used to analyze outcomes measured at one time point, as appropriate. Trajectories of change for outcomes that are repeatedly measured over time will be examined using generalized linear mixed models (GLMMs) and linear, logistic, and negative binomial/Poisson regression models for continuous, binary, and count outcomes. Total scores and subscales from assessments will be used when applicable. The use of GLMM models will allow for an estimation of between-group differences in outcomes at each time point of measurement and will allow for an estimation of changes over time and time by group interactions while accounting for within-subject associations arising from the repeated measures. Clustering of participants will be accounted for in the GLMMs models by including cluster as a random or fixed effect and in the generalized models by using robust variance estimates. Potential covariates will be selected based on consideration of study arm balance and potential confounders for an outcome previously reported in the literature.

The analyses described above will be repeated for the secondary outcomes. We will construct GLMMs for repeated outcomes (e.g., mixed-effects linear regression models for continuous outcomes and mixed-effects logistic regression models for dichotomous outcomes) and ANOVA and generalized linear or logistic regression models for outcomes measured at one time point.

All analyses will be two-tailed with an overall level of significance of 0.05. The Benjamini–Hochberg method will be used for adjusting the level of significance for multiple testing. The statistical software SAS, version 9.4, and the latest version of R will be used for data analysis.

#### Qualitative data

Thematic analyses will be conducted using an inductive approach, relying on the participant’s subjectively reported experiences and perceptions. Interview transcripts will be independently reviewed by an interdisciplinary team of coders led by expert qualitative researchers using open coding procedures. The codebook will be developed through an iterative process involving discussions between team members over code identification and application. A subset of interview transcripts will be coded independently by all members of the coding team to establish acceptable inter-coder reliability (pooled Cohen’s Kappa coefficient > 0.80), after which the remaining transcripts will be divided among the coders. Transcript excerpts will be organized into emergent key themes. Thematic saturation will be determined when no new themes are identified. Findings will be presented to a subset of participants to elicit feedback to validate thematic analyses. Debrief sessions will also be held with the research team to present findings and further validate or challenge interpretations of the data. Results will be reported in accordance with Consolidated Criteria for Reporting Qualitative Research Guidelines.

### Interim analyses {21b}

We will not conduct interim analyses.

### Methods for additional analyses (e.g., subgroup analyses) {20b}

Potential differences in treatment effects across influential participant characteristics (e.g., maternal age, number of children, infant sex) and group WCC characteristics (e.g., group size) will be assessed. A scale variable that will reflect a participant’s attendance level at group WCC visits will be created and included in the models to evaluate whether there is a “dose–response” intervention effect. Analyses will follow intent-to-treat principles, with participants analyzed according to randomized treatment assignment. In addition, a “per-protocol” analysis will be performed; the per-protocol analysis would include only participants who were adherent to their assigned study regimen and completed 75% of the recommended number of WCC sessions by 18 months of follow-up.

### Methods in analysis to handle protocol non-adherence and any statistical methods to handle missing data {20c}

The randomness of missing data between subjects and within given a subject will be investigated using available information on subject characteristics to help discern patterns in the missing data and to identify the possible missing data mechanisms (e.g., missing at random). Imputation strategies used to handle missing data will be considered as appropriate.

### Plans to give access to the full protocol, participant-level data and statistical code {31c}

Upon completion of the trial, the study protocol and de-identified participant-level data for own research purposes can be requested by submitting a research proposal to the principal investigators (Dr. Neera Goyal [Neera.Goyal@nemours.org] and Dr. Vanessa Short [vanessa.short@jefferson.edu]).

## Oversight and monitoring

The principal investigators will monitor the study to ensure that it is conducted according to the IRB-approved protocol.

### Composition of the coordinating center and trial steering committee {5d}

The trial steering committee consists of the principal investigators and all co-investigators. Team meetings occur on a monthly basis. This specified steering committee is responsible for overall oversight and governance of the trial with the final responsibility remaining with the PIs.

### Composition of the data monitoring committee, its role and reporting structure {21a}

Due to the low risk of the study, a formal data monitoring committee was not formed. There are no formal stopping rules for the trial because there are no anticipated problems that are detrimental to all participants. The principal investigators will review all data collection forms and electronic databases on an ongoing basis for data completeness and accuracy as well as protocol compliance. The research assistant will report weekly on recruitment, participants retained, and whether any issues with confidentiality or unexpected participant or data issues have arisen.

### Adverse event reporting and harms {22}

Monitoring and reporting of adverse events will be conducted from the time of consent to trial entry until the end of the trial. Adverse events and harms will be collected non-systematically, i.e., through spontaneous reporting of events. Research personnel will be asked to report unanticipated problems and adverse events, including serious adverse events such as an important medical event, hospitalization, or death of a mother or of a child of an enrolled mother, within 24 h of learning of the event. If known, the reason for the event should also be reported. The principal investigator, who is a pediatrician, will review the event within 24 h. The purpose of the review will be to assess if there is any reason that the event can be considered attributable to the study’s intervention. If the study is considered a causative factor, the report will be forwarded to the other principal investigator. The principal investigators will then determine the appropriate response through consultation with appropriate individuals. Adverse events will be documented in the participant file and reported to the appropriate IRB. It is believed that rarely would the study be considered a possible cause, but the establishment of the review procedure should assure that harm potentially attributable to the study is not missed and that any identified harm is addressed and avoided going forward. The review procedures developed for this study consist of weekly meetings with the opioid treatment program clinical team to review specific issues that were identified in the group visits. Issues such as maternal substance use or relapse, child safety concerns, and indications for child protective service involvement as well as housing, family safety, and other concerns are addressed with the team and clinical therapists, social workers, navigators, or peer support specialists are engaged to address issues.

### Frequency and plans for auditing trial conduct {23}

Regular meetings (weekly/monthly) will be held to monitor the study conduct and address potential problems.

### Plans for communicating important protocol amendments to relevant parties (e.g., trial participants, ethical committees) {25}

Changes to the protocol will be communicated to the IRB and approval from the IRB will be obtained prior to implementing any proposed changes.

### Dissemination plans {31a}

Trial results will be disseminated at local and national conferences and submitted for publication in peer-reviewed journals.

## Discussion

Given the current opioid epidemic and the increase in opioid use and misuse among pregnant women in the USA, this project is timely and highly relevant. The integration of group WCC into maternal OUD treatment represents a “game-changer” in disrupting health system silos and addresses the intergenerational effects of maternal OUD by improving parenting and engagement in and experience of preventative care. Findings from this work will be critical to informing implementation at a national scale.

## Trial status

Trial recruitment began on December 30, 2021. It is expected that recruitment will end in 2023.

## Data Availability

Following the completion of data entry, only the trial investigators and trial coordinators will have access to the final trial dataset.

## Abbreviations

OUD: Opioid use disorder
WWC: Well child care
